# Bacterial Community Shift in Treated Periodontitis Patients Revealed by Ion Torrent 16S rRNA Gene Amplicon Sequencing

**DOI:** 10.1371/journal.pone.0041606

**Published:** 2012-08-01

**Authors:** Sebastian Jünemann, Karola Prior, Rafael Szczepanowski, Inga Harks, Benjamin Ehmke, Alexander Goesmann, Jens Stoye, Dag Harmsen

**Affiliations:** 1 Department for Periodontology, University of Münster, Münster, Germany; 2 Institute for Bioinformatics, Center for Biotechnology, Bielefeld University, Bielefeld, Germany; 3 Bioinformatics Resource Facility, Center for Biotechnology, Bielefeld University, Bielefeld, Germany; 4 Genome Informatics Group, Faculty of Technology, Bielefeld University, Bielefeld, Germany; University of Hyderabad, India

## Abstract

Periodontitis, one of the most common diseases in the world, is caused by a mixture of pathogenic bacteria and inflammatory host responses and often treated by antimicrobials as an adjunct to scaling and root planing (SRP). Our study aims to elucidate explorative and descriptive temporal shifts in bacterial communities between patients treated by SRP alone versus SRP plus antibiotics. This is the first metagenomic study using an Ion Torrent Personal Genome Machine (PGM). Eight subgingival plaque samples from four patients with chronic periodontitis, taken before and two months after intervention were analyzed. Amplicons from the V6 hypervariable region of the 16S rRNA gene were generated and sequenced each on a 314 chip. Sequencing reads were clustered into operational taxonomic units (OTUs, 3% distance), described by community metrics, and taxonomically classified. Reads ranging from 599,933 to 650,416 per sample were clustered into 1,648 to 2,659 non-singleton OTUs, respectively. Increased diversity (Shannon and Simpson) in all samples after therapy was observed regardless of the treatment type whereas richness (ACE) showed no correlation. Taxonomic analysis revealed different microbial shifts between both therapy approaches at all taxonomic levels. Most remarkably, the genera *Porphyromonas*, *Tannerella*, *Treponema*, and *Filifactor* all harboring periodontal pathogenic species were removed almost only in the group treated with SPR and antibiotics. For the species *T. forsythia* and *P. gingivalis* results were corroborated by real-time PCR analysis. In the future, hypothesis free metagenomic analysis could be the key in understanding polymicrobial diseases and be used for therapy monitoring. Therefore, as read length continues to increase and cost to decrease, rapid benchtop sequencers like the PGM might finally be used in routine diagnostic.

## Introduction

Chronic periodontitis has a complex etiology and is an endemic inflammatory disease caused by a mixed bacterial infection with pathogens in intraoral biofilms. It is characterized by severe destruction of teeth supporting periodontal tissues, i.e., the connective tissue attachment apparatus and alveolar bone. Advanced forms of periodontitis may lead to edentulism in adulthood despite therapy and cause high costs for prosthetic rehabilitation. Several bacteria, mainly Gram-negative, are shown to be strongly associated with periodontal infections, e.g. *Porphyromonas gingivalis, Tannerella forsythia*, and *Treponema denticola* [1]. Of the about 700 species detected in the oral cavity based on culture and molecular analysis, more than half have not yet been cultivated [2], [3]. Approximately 400 of these species have been detected in subgingival pockets, although single patients harbor only a fraction of these species. Recently, next-generation sequencing techniques were used to investigate oral samples from healthy individuals enabling for a deeper coverage of the microbial community [4], [5].

Mechanical debridement, i.e. the removal of biofilms from tooth surfaces by scaling and root planing (SRP) is the established gold standard in the treatment of periodontal disease [1] but adjunctive systemic antibiotics are frequently used as supportive therapeutic agents. Two systematic reviews about the impact of various adjunctive antibiotic agents in periodontal therapy conclude that antibiotics may offer a clinical benefit [6], [7]. However, it is insufficiently studied which periodontitis patients may profit from antibiotic treatment, what the preferred treatment interval might be, and how antibiotics affect the whole subgingival microflora. Therefore, our study aims to elucidate explorative and descriptive temporal shifts in bacterial communities as analyzed by next-generation sequencing between patients treated by SRP alone versus SRP with adjunctive antibiotics. To explore richness and diversity of subgingival plaque samples the hypervariable region V6 of the 16S rRNA gene was targeted. By using the fast and per experiment rather affordable Ion Torrent’s Personal Genome Machine (PGM) a platform was applied that eventually might also be used in routine diagnostics.

## Materials and Methods

### Patients, Sample Collection and DNA Isolation

In total, samples from four patients were taken before (pre) and two months after (post) intervention. Patients were part of a double-blind, parallel group, randomized, placebo-controlled multi-center efficacy study (ISRCTN64254080). The study was approved by the Medical Ethical Committee of the University Münster (ref: 2006-474-f-A) and a signed written informed consent was obtained from each patient. The four selected patients fulfilled the study’s inclusion criteria and were part of one stratum (non-smokers, generalized severe chronic periodontitis, i.e. more than 38% of sites with pocket probing depths of 6 mm or more). The intervention consisted either of mechanical debridement plus 500 mg amoxicillin and 400 mg metronidazole three times daily for 7 days (experimental, Ex; two patients) or mechanical debridement alone (control, Co; two patients). For microbiological analysis, subgingival plaque samples from each patient were pooled. Specimens were taken from four different teeth with at least one site having a pocket probing depth (PPD) of ≥6 mm. Sample teeth were randomly selected before intervention to assure equal distribution throughout the mouth. At the four sample sites supragingival plaque was gently removed, teeth were air-dried and isolated with cotton rolls. One sterile paper point (ISO45, Roeko Dental, Langenau, Germany) was inserted for 10 seconds in each site and all paper points were removed and pooled in one transport tube. Samples were stored at −20°C until further use. Bacterial genomic DNA was isolated and purified with the QiaAmp Mini DNA-Isolation Kit (Qiagen, Hilden, Germany). The protocol followed the manufacturer’s instructions with minor modifications, i.e., a pretreatment step with proteinase K (20 mg/ml) at 56°C was carried out overnight. After isolation, the purified DNA was eluted in 200 µl of elution buffer. Quality and purity of the isolated genomic DNA was confirmed by agarose gel-electrophoresis and spectrophotometry on the NanoDrop 2000 device (Fisher Scientific, Schwerte, Germany). DNA concentration was estimated with the Qubit 2.0 instrument applying the Qubit dsDNA HS Assay (Life Technologies, Invitrogen division, Darmstadt, Germany).

### 16S Primers and Amplicon Library Generation

PCR amplification of the 16S rRNA hypervariable region V6 was performed with a pool of six degenerated forward and six degenerated reverse primers targeting bacteria and archaea as described by Huber at al. [8]. The 5′-ends of the forward primers were fused with the A-Adaptor plus key sequence, whereas the reverse primers were fused with the truncated Pi-adapter sequence (trP1), respectively. The primers were diluted in molecular grade laboratory water and pooled equimolar. For amplicon library preparation 4 ng of each genomic DNA, 1 U Platinum Taq DNA polymerase High Fidelity, 5 mM dNTPs, 2 mM MgCl_2_ (all Life Technologies), and 10 pmol primer-mix were used per 25 µl amplification reaction. The PCR conditions were as follows: 94°C for 3 min, followed by 36 cycles of 94°C for 15 s, 58°C for 15 s, 68°C for 10 s, and a final elongation step of 68°C for 30 s. Amplicon product purification was performed via gel-electrophoresis on a 1.5% Tris Borat EDTA agarose gel [9] stained with SybrGold (Life Technologies) using the peqGold S gel extraction kit (Peqlab Biotechnologie GmbH, Erlangen, Germany). Control of purification and determination of exact fragment sizes was carried out with the Caliper GX system using the HT DNA High Sensitivity LabChip Kit (Caliper Life Sciences GmbH, Mainz, Germany). Amplicon library concentration was estimated with the Qubit 2.0 instrument using the Qubit dsDNA HS assay (Life Technologies). From the concentration and the average size of each amplicon library, the amount of DNA fragments per micro liter was calculated and libraries were diluted to 2.8×10^8^ DNA molecules per micro liter prior to clonal amplification.

### Emulsion PCR and Sequencing

The emulsion PCR was carried out applying the Ion XPress Template kit V2.0 (Life Technologies) as described in the appropriate user Guide (Part No. 4469004 Rev. B 07/2011) provided by the manufacturer. Quality and quantity of the enriched spheres were checked on the Guava easyCyte5 system (Millipore GmbH, Schwalbach am Taunus, Germany) as described in the appendix of the Ion Xpress Template Kit User Guide (Part Number 4467389 Rev. B, 05/2011). Sequencing of the amplicon libraries was carried out on the Ion Torrent Personal Genome Machine (PGM) system using the Ion Sequencing 200 kit (all Life Technologies) following the corresponding protocol (Part No. 4471999 Rev B, 13. Oct. 2011) with minor modifications: i) the chip was washed one more time with isopropanol and annealing buffer after chip check and calibration in order to remove possible air bubbles introduced during these procedures; ii) the spheres were loaded twice onto a 314 chip, each loading was followed by four rounds of centrifugation at maximum speed for 15 s (Mini Star, VWR International GmbH, Darmstadt, Germany), shaking on the IKA orbital shaker (IKA-Werke GmbH & Co. KG, Staufen, Germany) equipped with a special chip adapter at 3000 rpm for 10 s, and each loading was finished by a final 15 s centrifugation step; and iii) the complete sample was loaded. For every sample a separate 314 chip (Life Technologies) was used. Raw sequence reads of all samples were deposited at the EBI Short Read Archive (SRA) and can be accessed under the study accession number ERP001440.

### Quantification of Selected Periodontal Pathogens

Absolute quantification of *P. gingivalis* and *T. forsythia* in the investigated subgingival plaque samples was achieved by real-time PCR. Genomic DNA of pure cultures of the two species was used to generate standard curves with known amounts of genomic equivalents. Primers and TaqMan probes targeting the 16S rRNA gene were used. Amplification of *P. gingivalis’* 16S rRNA gene was performed in 20 µl reactions composed of 8 µl 2.5× Real Mastermix Probe (5Prime, Hamburg, Germany), 200 nM forward primer (Pg-146f 5′-TGCAACTTGCCTTACAGAGGG-3′), 250 nM reverse primer (Pg-447r 5′-ACTCGTATCGCCCTGTATTC-3′), and 125 nM TaqMan probe (Pg-195f-FAM 5′-FAM-CGGACTAAAACCGCATACACTTGTATTATTG-BHQ1-3′). Amplification of *T. forsythia’s* 16S rRNA gene was done accordingly with 300 nM forward primer (Tf-133f 5′-GGGTGAGTAACGCGTATGTAACCT-3′), 300 nM reverse primer (Tf-212r 5′-ACCCATCCGCAACCAATAAATC-3′), and 125 nM TaqMan probe (Tf-157f-FAM 5′-FAM-CCCGCAACAGAGGGATAACCCGG-BHQ1-3′). To each reaction 2 µl of the subgingival plaque DNA was added. All samples were run in duplicates on a MasterCycler realplex^4^S (Eppendorf, Hamburg, Germany). The PCR conditions were as follows: 95°C for 2 minutes, followed by 40 cycles of denaturation at 95°C for 15 s, and a combined annealing/elongation step at 60°C for 60 s.

### Sequence Analysis

Raw sequencing reads were consecutively checked for different quality criteria. Low quality reads were removed as follows: i) reads with no match against the trP1 adapter allowing for up to three errors; ii) reads not matching the degenerated PCR primers with at most two errors; iii) sequences containing ambiguously called bases (N); and iv) reads with an average quality score below the 5^th^ percentile of all reads (95 percent confidence interval). Correctly detected primer sequences were trimmed off during this procedure. Primer matching and trimming were performed by extensions to the Conveyor workflow engine [10] in combination with Cutadapt [11], requiring a minimum primer overlap of 10 bases and penalizing initial gaps in the semi-global alignment. Derived high quality sequences were screened for artificial chimeric formations using the UCHIME algorithm [12]. In order to de-noise sequencing data, reads were further applied to the Single Linkage Preclustering (SLP) method [13] implemented in mothur [14]. In this process, sequences having a pairwise distance less than 2% were clustered using a modified single linkage algorithm and merged while retaining cluster sizes as counts. Following analyses were performed using only high quality reads de-noised by SLP.

Subsequently, operational taxonomic unit (OTU) analysis was done on a clustering basis for each sample individually. Reads were clustered at 97% identity threshold using the complete linkage clustering algorithm implemented in ESPRIT [15]. To meet the known problem of species overestimation [16], reads from singleton OTUs were excluded based on results of another upstream clustering, as recommended [17]. Rarefaction curves and the species richness estimators ACE [18] were calculated by ESPRIT. The abundance accounting Shannon and Simpson diversity indices [19] were computed using the statistical software suite R v 2.9.10 [20] and the vegan R-package [21]. Finally, assignment of taxonomic annotation to high quality reads was achieved using the GAST [22] pipeline with default parameters in combination with the SILVA rRNA database (version 102) [23]. In brief, GAST compares each sequence to a reference database, selects the closest reference(s), and assigns the taxonomy based on the lowest common ancestor for a two-third majority of the references. A schematic overview of the experimental work-flow and applied bioinformatic procedure is given in **Figure S1**.

## Results and Discussion

Next-generation sequencing technologies extended our understanding of microbial communities and were applied in a vast number of metagenomic studies. The recently introduced Ion Torrent PGM constitutes a new type of next-generation benchtop sequencing platform. Main advantages of this machine are the small size, low acquisition price, and fast turnaround time. Here, we used the PGM to analyze subgingival plaque samples of four patients for shifts in the microbial community in response to treatment by SRP and SRP with adjunctive antibiotics. Demographic data, baseline and post intervention clinical parameters (pocket probing depth, bleeding on probing (BOP), and plaque index (PI)) are listed in **Table S1**. All clinical parameters improved after intervention in the control and experimental group markedly, i.e. shallow pockets (< = 3 mm) increased whereas initially deep sites decreased. No obvious differences between both groups could be observed. Due to limited sample size and short follow-up statistical inference was not performed. However, it is documented in numerous studies and systematic reviews that the administration of adjunctive antibiotics result in further improvements of clinical parameters in comparison to SRP treatment alone e.g. [6], [7], [24], [25].

For the 16S rRNA gene amplicon sequencing we targeted the V6 hypervariable region, corresponding to positions 984–1047 (*Escherichia coli*). Although being a rather short region, it has been shown to enable successful discrimination between different phylotypes [26]. By using the Silva database it was demonstrated in an *in-silico* analysis that more than 97% of V6 tags could be unambiguously mapped at genus level [22]. In addition, V6 was not only the hypervariable 16S rRNA gene region used in the first NGS metagenomic amplicon study [27] but is still most frequently applied in environmental and medical microbiology projects e.g. [4], [28]. Furthermore, amplicons of single hypervariable regions missing conserved intermediate stretches and short targets in general are less prone to form chimeras, therefore contributing to higher data quality [29]. In total, 4,892,600 sequence reads, ranging from 599,933 to 650,416 per sample and 314 chip were generated on the Ion Torrent PGM machine (**Table 1**). Downstream sequence processing and quality filtering removed about 12.5% of the raw sequencing data. Most sequences were removed from further analysis due to untraceable sequencing or amplification primers (∼8%) and significant low quality scores (∼4.5%). Chimeric sequences were found only in very small quantities (less than 1% per sample). Sequences of each data set were further de-noised by SLP, by which between 9% and 14% of all reads were compensated for potential sequencing errors resulting in 5,210 to 8,462 unique tag sequences per sample (**Table 1**).

**Table 1:** Summary of sequence processing.

Sample$	Total reads	High-quality &non-chimeric reads*	De-noised uniquereads**
Co-pre-1	613,943	537,150	7,789
Co-post-1	627,905	551,840	5,555
Co-pre-2	620,533	552,247	6,325
Co-post-2	612,292	550,532	8,462
Ex-pre-1	602,209	528,664	5,210
Ex-post-1	599,933	505,452	6,596
Ex-pre-2	565,369	492,048	8,552
Ex-post-2	650,416	566,915	7,144

$ Sample abbreviations: Co, control group; Ex, experimental group; pre, before intervention; post, after intervention.

* Number of remaining reads after applying quality criteria as described in the Methods section.

** Unique high quality reads after de-noising the data by Single Linkage Preclustering at 2% edit distance.

**Figure 1:**
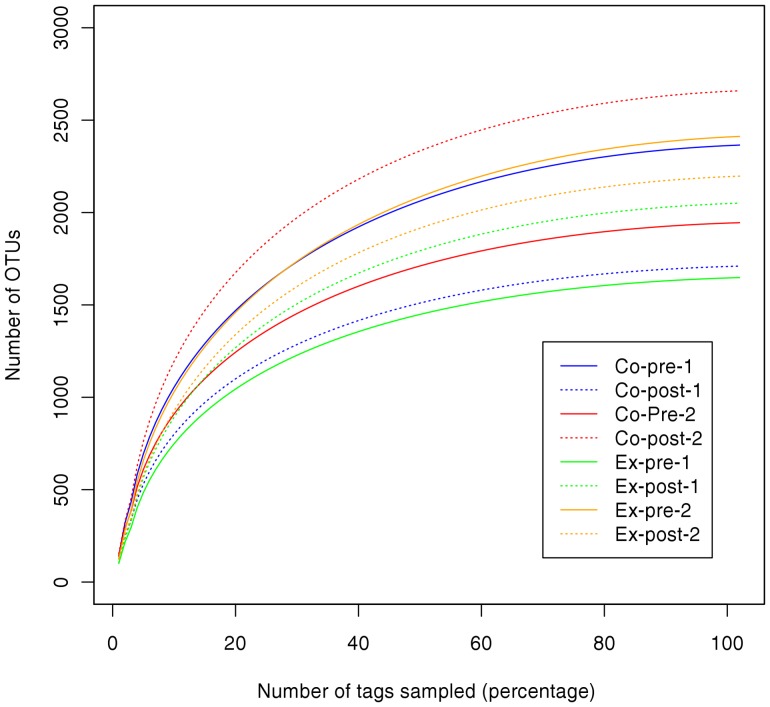
Rarefaction analysis for each sample based on clustering of high quality de-noised reads. In total samples from four patients were analyzed two times, i.e., before (continuous lines) and two months after intervention (dashed lines). Two control group patients (Co) were treated by SRP alone whereas two experimental group patients (Ex) received SRP plus adjunctive antibiotics. Lines are colored according to the sample origin.

Clustering of de-noised high quality reads at species-level, defined by 3% sequence difference, generated between 3,499 and 6,058 OTUs per specimen (**Table 2**). Exclusion of singleton OTUs prior to computation of community metrics decreased OTU counts to about 50% while affecting only 0.5% of the sequencing data. Non-singleton OTUs ranged from 1,648 to 2,659 counts per sample. ACE species richness estimations were almost identical to the OTU numbers demonstrating a sufficient sampling effort (**Table 2**). This is furthermore supported by rarefaction analysis where all curves almost reached saturated plateau phase (**Figure 1**). No correlation between the number of OTUs and sampling time or treatment type could be observed. Our phylotype estimates were about a log order higher than those determined by cloning and traditional Sanger sequencing [2]. When analyzing pooled supragingival plaque samples from 89 healthy adults by 454 pyrosequencing, Keijser *et al.* reported 10,052 OTUs [4] at 3% difference. The first metagenomic study using Illumina’s high-throughput sequencing technology by Lazarevic *et al.* [5] inferred about 8,000 different phylotypes at 3% OTU distance from pooled oral samples of three healthy adults. In contrast, Griffen *et al.* [30] found between 100 and 300 species OTUs in single individuals when analyzing subgingival samples of 29 subjects with chronic periodontitis by 454 pyrosequencing. However, in their study OTUs were not defined by hierarchical clustering but based on similarity to sequences of a curated oral 16S rDNA database [31]. This demonstrates that OTU profiles and metrics based thereupon are highly dependent on data characteristics and applied processing methods. However, community comparisons by species richness and diversity estimates are still meaningful if data were generated and analyzed in the same way. In our study, Shannon and Simpson indices showed a consistent pattern, i.e. increased diversity in all samples after therapy regardless of the treatment type, with sample Ex-Pre-1 representing the least (Shannon: 3.68; Simpson: 0.11) and Co-Post-2 the most diverse (Shannon: 4.91; Simpson: 0.01) community (**Table 2**). As diversity also considers OTU abundances, our results reflect more evenly distributed abundance profiles after treatment, i.e. prevalent organisms of a mature biofilm were suppressed by therapy inducing favorable growing conditions for rare and new species. This is also supported by Pielou’s evenness values that were indicating more equally distributed OTU abundances among community members in post-treatment samples. It is interesting to note that Kumar at *al*. when comparing subjects with healthy implants and peri-implantitis using 16S pyrosequencing found a significant increase in species diversity in the healthy subgroup [32].

**Table 2:** OTU counts, richness and diversity estimates for the bacteria in subgingival plaque samples before and after intervention.

Sample	OTUs*	ACE estimator	Shannon index	Simpson index	Pilous evenness
Co-pre-1	2,365 (5,273)	2,421	4.36	0.05	0.56
Co-post-1	1,710 (3,848)	1,749	4.45	0.02	0.59
Co-pre-2	1,945 (4,377)	1,989	4.42	0.04	0.58
Co-post-2	2,659 (5,705)	2,718	4.91	0.01	0.62
Ex-pre-1	1,648 (3,499)	1,691	3.68	0.11	0.49
Ex-post-1	2,051 (4,449)	2,100	4.03	0.05	0.53
Ex-pre-2	2,412 (6,058)	2,476	4.19	0.05	0.54
Ex-post-2	2,197 (5,051)	2,250	4.38	0.03	0.60

* OTU clusters were generated by complete linkage clustering at 97% identity threshold excluding singleton clusters; numbers in parentheses give OTU counts including singleton clusters.

**Figure 2:**
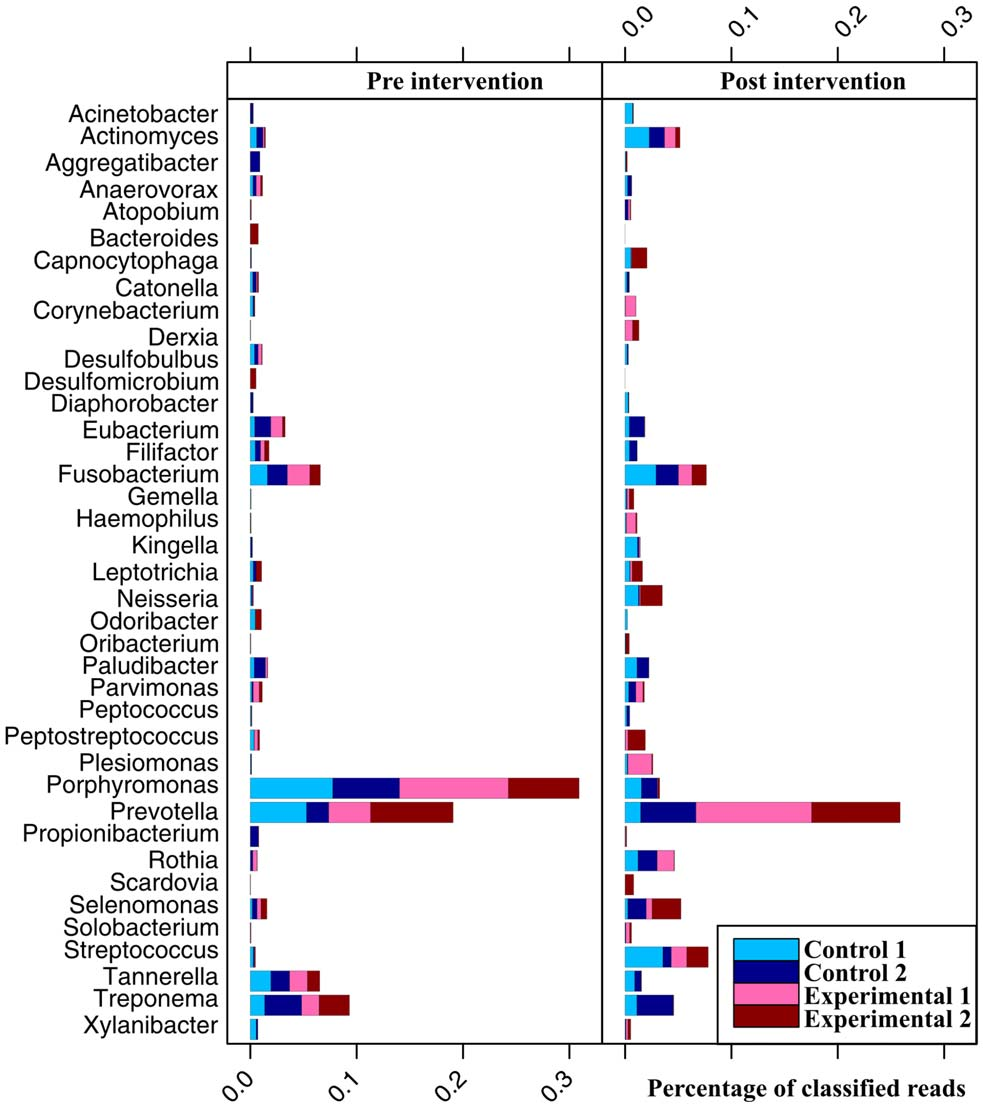
Relative abundance of the most prevalent genera (> = 1% relative abundance within one sample) in subgingival plaque samples before and after intervention. Each bar displays the normalized relative abundance (individual relative abundance divided by the number of analyzed samples at the given time point) of the corresponding genera pre and post intervention. Each bar is further partitioned into four stacked parts for each of the four analyzed patients. The color indicates the type of intervention (blue for control, red for experimental group).

Almost all community members in our samples originated from the bacteria domain. The most prevalent phyla in all samples were: *Bacteroidetes*, *Firmicutes*, *Fusobacteria*, *Proteobacteria*, *Actinobacteria*, *Spirochaetes*, and *Synergistetes* (**Table 1**) comprising together more than 99.8% of all reads. Irrespective of treatment type a shift from Gram-negative *Bacteroidetes* to Gram-positive *Firmicutes* was observed after treatment. At genus level, sequencing reads could be assigned to 330 individual genera, of which 38 were present in all samples with a relative abundance of more than one percent in at least one sample (**Figure 2**). On average, in eight percent of all cases a successful classification at genus level was not possible, resulting in a classification at a higher taxonomic rank (data not shown).

Specimens taken prior to any treatment showed a highly similar community composition by means of taxonomic groups and abundance proportions. *Bacteroidetes*, *Firmicutes*, *Fusobacteria*, and *Spirochaetes* were the most prevalent phyla (**Table 3**), which is in accordance with previous studies [2], [4], [5]. At genus level the predominant genera were *Porphyromonas*, *Prevotella*, *Treponema, Fusobacterium*, and *Tannerella* (**Figure 2**), occupying about 67% of the community sequences. Among these, three genera are of particular interest, as they include the species *Porphyromonas gingivalis, Treponema denticola*, and *Tannerella forsythia*, proposed to form the pathogenic ‘red complex’ consortium in periodontitis [33]. Furthermore, the genus *Prevotella* also includes several known periodontitis associated bacteria (*Prevotella nigrescens*, *Prevotella intermedia*, *Prevotella melaninogenica*) [33], [34]. Other genera related to pathogenic organisms could also be identified, but either only in specific samples (e.g. *Aggregatibacter*) or at lower abundances (e.g. *Parvimonas*).

**Table 3:** Relative abundance of the main phyla identified in subgingival plaque samples.

	Sample							
	Control				Experimental			
	pre		post		pre		post	
Phylum$	1	2	1	2	1	2	1	2
*Actinobacteria*	3.70	7.15	12.61	13.52	2.44	1.02	12.58	5.57
*Bacteroidetes*	63.53	44.75	21.43	35.34	63.11	61.45	36.72	40.07
*Firmicutes*	14.68	14.88	26.36	25.9	16.37	13.22	14.50	30.76
*Fusobacteria*	6.99	8.19	12.11	8.02	8.27	11.11	4.72	9.08
*Proteobacteria*	4.65	10.96	22.86	4.05	2.67	2.57	31.36	14.45
*Spirochaetes*	5.04	13.24	3.98	12.89	6.42	10.11	0.09	0.04
*Synergistetes*	0.84	0.71	0.25	0.19	0.67	0.42	0.0004	0.0009
Other*	0.32	0.0994	0.2667	0.0705	0.0358	0.0193	0.0255	0.0111
Unclassified**	0.25	0.0147	0.1455	0.0064	0.0013	0.0790	0.0093	0.0089

$ Only phyla with a relative abundance of more than half a percent within at least a single sample are shown.

* All remaining phyla with relative abundance lower than half a percent.

** Reads where unambiguous taxonomic classification was not possible.

For post-treatment samples the three most prevalent genera were *Prevotella*, *Streptococcus*, and *Fusobacterium* constituting about 37 percent of the sequences. Specimens of the experimental group showed markedly decreased abundances of the genera *Treponema* and *Filifactor* with frequencies dropping down to complete absence for *Filifactor* in one patient. In the control group these genera remained relatively stable. The genera *Porphyromonas* and *Tannerella* were suppressed in both groups. However, in the experimental group they were virtually absent (**Figure 1**). Due to the limited read length of most next-generation sequencing platforms, taxonomic resolution down to species level is impossible. Therefore, we additionally screened all samples for *P. gingivalis* and *T*. *forsythia* using species-specific real-time PCR. A positive treatment effect indicated by a reduction of genomic equivalents of both species could be observed irrespectively of the treatment type (**Table 4**). However, in the experimental group *P. gingivalis* and *T. forsythia* were also suppressed to virtual absence, thereby corroborating our NGS data. Our observed bacterial community shifts are in accordance with Valenza *et al.* [35], who did a longitudinal capillary sequencing rDNA cloning study and reported that two months after treatment with SRP plus amoxicillin and metronidazole, disease associated genera were suppressed (e.g. *Treponema*, *Porphyromonas*, *Filifactor*, *Tannerella*). However, these therapy effects were reversible after twelve months. Based on cultivation and on species specific PCR assays the very same effect of systemic adjunctive antibiotics on *P. gingivalis* and *T. forsythia* was previously reported [24], [25].

**Table 4:** Genomic equivalents per microliter of *P. gingivalis* and *T. forsythia* detected by real-time PCR.

	Sample							
	Control				Experimental			
	pre		post		pre		post	
Phylum	1	2	1	2	1	2	1	2
*P. gingivalis*	338,000	45,499	6,450	2,326	244,000	600,000	n.d.*	n.d.
*T. forsythia*	98,500	18,601	6,929	10,026	64,500	66,500	1	14

* not detected

In our study several genera had increased abundance values in the control and experimental group after treatment, i.e. *Prevotella*, *Selenomonas*, *Streptococcus*, *Actinomyces,* and *Rothia*. Of them, *Streptococcus*, *Actinomyces,* and *Rothia* comprise known early colonizer species [36]. The finding that the antibiotic treatment seemed to have selectively eradicated some genera such as *Porphyromonas* while other genera such as *Prevotella* were not affected is difficult to explain. In principle the effects might be due to different recolonization dynamics or antibiotic susceptibility. Since taxonomic resolution down to species-level is impossible we were not able to determine if the sequencing reads counting for the genus *Prevotella* before and after treatment belonged to the same species. *Prevotella loescheii* is known to be an early colonizing partner of receptor-bearing streptococci (first colonizers) [36] therefore a shift from periodontitis associated *Prevotella* species towards the early colonizing species could be conceivable. It is known that the gingival crevicular concentration of metronidazole and amoxicillin is at least ten times the *in-vitro* minimal inhibitory concentration (MIC_90_) of *P. gingivalis*, *P. intermedia* and *P. nigrescens* [37]. However, some *Prevotella* species might have developed active resistance mechanisms, e.g. Bernal *et al.* reported that about 26% of *P. intermedia*- and *P. nigrescens* isolates are beta-lactamase positive and thereby at least not susceptible to amoxicillin [38]. In addition to the genera *Selenomonas* and *Streptococcus,* Valenza *et al.* [35] also observed for *Prevotella* an increase in abundance early after intervention. In contrast to them we were able to relate effects after treatment either to antibiotic or mechanical debridement therapy by inclusion of a control group.

Whether the detection of selected periodontal pathogens is useful for treatment decision remains questionable. For example, by testing for *A. actinomycetemcomitans*, *Fusobacterium nucleatum spp.*, *P. gingivalis*, *P. intermedia*, *T. denticola*, and *T. forsythia* Cionca *at al.* showed that it was of no predictive value in identifying subjects who would benefit from adjunctive antibiotic treatment [39]. In another recent study the authors suggested a correlation of these species with pocket probing depth rather than periodontal diagnosis [40]. Therefore, analyzing hypothesis free the complete microbial community by NGS might be the key in understanding a chronic disease like periodontitis, as the ‘pathogen’ might be a disturbed microbial community rather than single organisms [41]. If the in this study observed differences in community profiles and species diversity of periodontitis patients after treatment implies a diagnostic useful prognostic factor for treatment success warrants further experiments with larger sample size, longer follow-up, and a treatment failure subgroup. As performance characteristics like reading length and error rate of the PGM further improves, more specific, e.g. by sequencing longer stretches of adjacent hypervariable regions [31], and less error prone descriptions of the microbial communities will be possible [42].

In conclusion, this translational metagenomic study gives new insights into differences of the subgingival microflora of periodontitis patients for two different treatment regimes. The study results indicate that changes in community profiles and metrics are potentially more diagnostically predictive for treatment success than the detection of selected periodontal pathogens. Monitoring changes in the subgingival microflora with platforms like the PGM might finally pave the way towards routine personalized medicine.

## Supporting Information

Figure S1 **Schematic overview of the applied experimental and bioinformatic steps throughout the analysis.** For a more detailed description of each individual step please see the Methods section.
(PDF)

Table S1 Demographic patient data and clinical parameters at baseline and post intervention.
(PDF)
