# Supplementary material for: Bacterial Community Shift in Treated Periodontitis Patients Revealed by Ion Torrent 16S rRNA Gene Amplicon Sequencing
Source: PLoS One. 2012 Aug 1;7(8):e41606. doi: 10.1371/journal.pone.0041606 (PMC3411582; doi:10.1371/journal.pone.0041606)
Supplement: Figure S1 — Schematic overview of the applied experimental and bioinformatic steps throughout the analysis. For a more detailed description of each individual step please see the Methods section. (PDF) [file pone.0041606.s001.pdf]

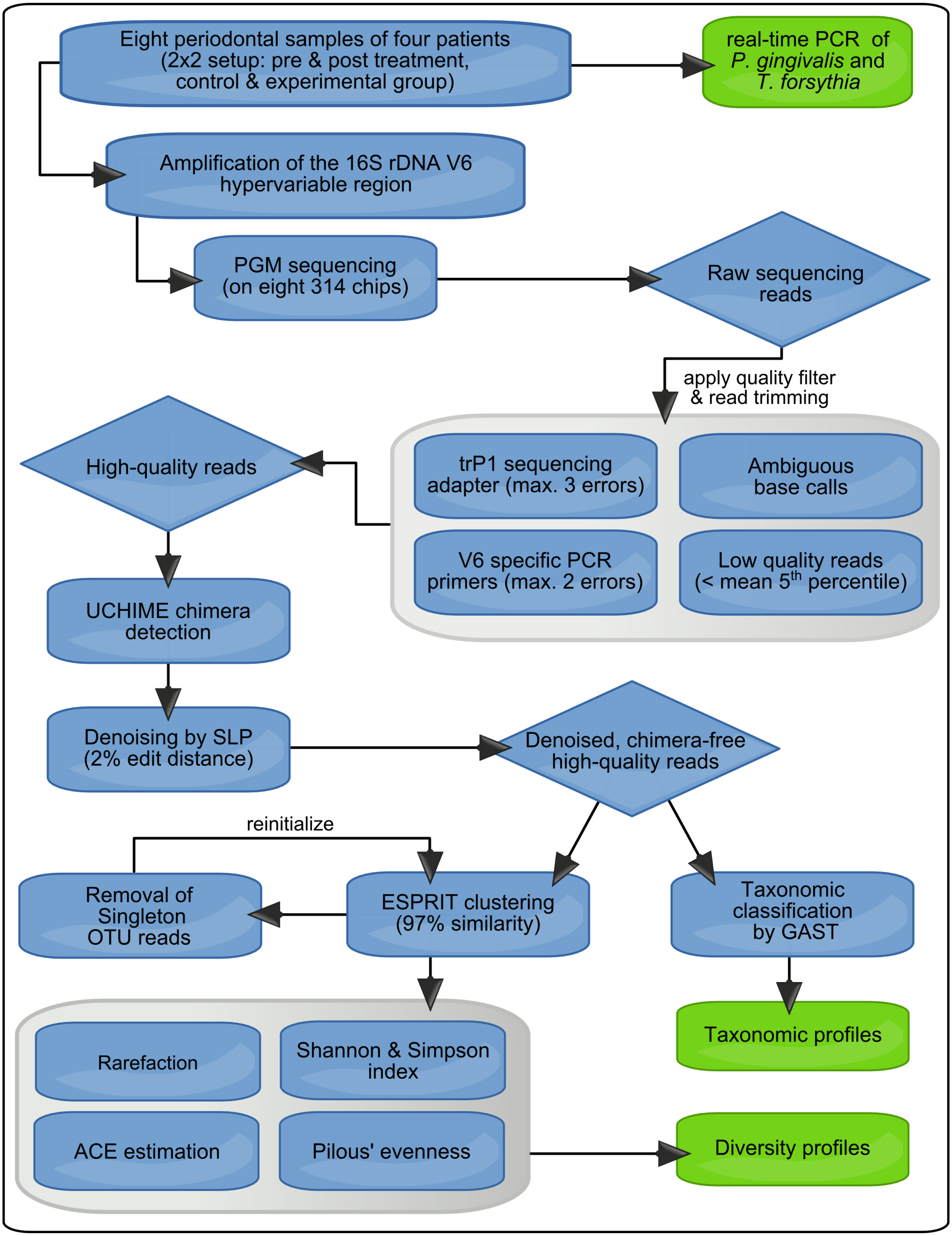

Figure S1. Schematic overview of the applied experimental and bioinformatic steps throughout the analysis. For a more detailed description of each individual step please see the Methods section.
