# Supplementary material for: Bacterial Community Shift in Treated Periodontitis Patients Revealed by Ion Torrent 16S rRNA Gene Amplicon Sequencing
Source: PLoS One. 2012 Aug 1;7(8):e41606. doi: 10.1371/journal.pone.0041606 (PMC3411582; doi:10.1371/journal.pone.0041606)
Supplement: Table S1 — Demographic patient data and clinical parameters at baseline and post intervention. (PDF) [file pone.0041606.s002.pdf]

Table S1. Demographic patient data and clinical parameters at baseline and post intervention

	Patient[$]			
	**Control-1**	**Control-2**	**Experimental-1**	**Experimental-2**
**Age (years)**	65	45	42	64
**N teeth***	28	28	24	15
**% sites with PPD** ≤ 3 mm (pre / post intervention)**	19.6 / 34.5	23.9 / 45.2	36.8 / 56.9	14.4 / 47.7
**% sites PPD 4-6 mm (pre / post)**	55.4 / 54.7	46.4 / 48.3	27.1 / 34.1	51.2 / 45.6
**% sites PPD ≥ 7 (pre / post)**	25 / 10.8	29.7 / 6.5	36.1 / 9	34.4 / 6.7
**% sites with BOP*** (pre / post)**	79 / 12	54 / 11	59 / 7	84 / 14
**% sites with plaque index (pre / post)**	73 / 12	52 / 21	50 / 5	98 / 37

[$] All patients were males, non-smokers with generalized severe chronic periodontitis - i.e. more than 38% of sites with PPD ≥ 6 mm - and with no professional periodontal therapy during the six months preceding the baseline clinical evaluation.

* For every tooth clinical parameters (PPD, BOP, plaque index) were determined from six sites.

** Pocket probing depth.

*** Bleeding on probing.
